# Trypanocidal Activity of Crude Extracts from *Montagnula* sp., an Endophytic Fungus Isolated from *Lippia alba*

**DOI:** 10.3390/jof12070536

**Published:** 2026-07-20

**Authors:** Karen Alexandra Caicedo-Jiménez, Liliana Torcoroma García, Erika Marcela Moreno, Catalina Salgado-Salazar, Julie Fernanda Benavides Arévalo, Beatriz Elena Guerra-Sierra

**Affiliations:** 1 Posgraduate Department in Infectious Disease, Facultad de Ciencias Médicas y de la Salud, Universidad de Santander, Bucaramanga 680006, Colombia; kar.caicedo@mail.udes.edu.co (K.A.C.-J.); l.torcoroma@udes.edu.co (L.T.G.); coord.maestriainfecciosas@udes.edu.co (E.M.M.); 2 Mycology and Nematology Genetic Diversity and Biology Laboratory, Agricultural Research Service, United States Department of Agriculture (USDA-ARS), Beltsville, MD 20705, USA; catalina.salgado@usda.gov; 3 Faculty of Health, Industrial University of Santander, Bucaramanga 680002, Colombia; jfbenare@uis.edu.co; 4 Faculty of Natural Sciences, Universidad de Santander, Bucaramanga 680006, Colombia

**Keywords:** Chagas disease, *Trypanosoma cruzi*, trypanocidal activity, *Montagnula* sp., endophytic fungi, *Lippia alba*, bioactive metabolites

## Abstract

Chagas disease, caused by *Trypanosoma cruzi*, remains a neglected tropical infection with limited therapeutic options and significant drug-related toxicity. Endophytic fungi are emerging as sustainable sources of bioactive metabolites with potential antiparasitic activity. In this study, endophytic fungi isolated from the medicinal plant *Lippia alba* were screened for trypanocidal activity. Crude extracts from seven isolates were tested against epimastigote and amastigote forms of *T. cruzi*, together with cytotoxicity assays in J774A.1 murine macrophages. The crude extract of *Montagnula* sp. exhibited the strongest trypanocidal activity (IC_50_ = 22.1 ± 0.5 μg/mL for epimastigotes and 29.2 ± 3.0 μg/mL for amastigotes), comparable to benznidazole, with low toxicity (CC_50_ > 600 μg/mL) and a high selectivity index (>26). Morphological and mitochondrial analyses demonstrated preservation of host-cell integrity and mitochondrial function, in contrast to the reference drug. UHPLC-ESI-HRMS profiling identified phenolic and terpenoid compounds, including caffeic acid, ferulic acid, naringenin, apigenin, and ursolic acid, which may underlie the observed biological activity. To our knowledge, this study provides the first evidence of trypanocidal activity in an endophytic Montagnula species and highlights endophytic fungi as promising platforms for the discovery of novel anti-*T. cruzi* agents.

## 1. Introduction

Chagas disease (CD) is a potentially fatal, zoonotic, and multisystem infection caused by the hemoflagellate protozoan *Trypanosoma* cruzi [1]. Although historically confined to 21 Latin American countries, CD is now also recognized as endemic in the United States [2]. In these endemic countries, nearly 90% of reported cases are concentrated, primarily associated with vectorial transmission by hematophagous triatomine insects. In contrast, in non-endemic regions, transmission occurs mainly through congenital infection, blood transfusion, and organ transplantation, with population mobility and the absence of systematic surveillance for *T. cruzi* acting as key driving factors [3]. Consequently, CD has become a global public health problem, with an estimated 7 million people infected worldwide [3].

Chronic chagasic cardiomyopathy (CCC) is the most severe and prevalent complication, affecting 30–50% of seropositive individuals [4]. This condition is characterized by progressive cardiac dysfunction, arrhythmias, heart failure, and sudden death, resulting in high morbidity, reduced quality of life, and substantial healthcare costs [5]. Current treatment relies on benznidazole (BNZ) and nifurtimox (NFT), which are effective in the acute phase but show limited efficacy in chronic infections, require prolonged regimens and are associated with significant toxicity, and variable susceptibility among parasite strains [6,7].

Natural products from medicinal plants have gained attention as alternative sources of antiparasitic agents. Essential oils (EOs) from medicinal plants such as *Lippia alba* (Mill.) N.E.Br. exBritt & Wils. (Verbenaceae) exhibit notable antimicrobial and trypanocidal activity, as well as cardioprotective effects in experimental models of chagasic cardiomyo-pathy [8]. However, large-scale application is hindered by variability in chemical composition, influenced by genotype, environment, seasonality, and extraction methods [9]. This underscores the need for more sustainable sources of bioactive metabolites.

Endophytic fungi, particularly Ascomycota, colonize plant tissues asymptomatically and are prolific producers of structurally diverse secondary metabolites, including terpenes, alkaloids, polyketides, phenolics, and glycosides, which play important roles in plant defense, stress tolerance, and ecological adaptation [10,11,12]. Endophytic fungi have demonstrated the ability to biosynthesize secondary metabolites that are chemically identical or functionally analogous to those produced by their host plants making them promising candidates for sustainable drug discovery and biotechnological production [11,12,13]. Previous studies have demonstrated antiparasitic activity of endophytic fungi against *T. cruzi* and *Leishmania donovani*, yet their diversity and metabolite profiles remain under explored, particularly in tropical medicinal plants [10,14,15,16].

The genus *Montagnula* (Didymosphaeriaceae, Dothideomycetes) comprises ecologically versatile species, such as M. *donacina*, which have been reported as saprobic and endophytic, colonizing a wide range of plant substrates in different ecosystems. Although the genus remains poorly explored, previous studies have reported antimicrobial and antiosteoclastogenic activities associated with secondary metabolites produced by *Montagnula* 7 8 species, highlighting their potential pharmacological relevance [17,18].

Here, we hypothesized that *L. alba* endophytic fungi produce secondary metabolites that could be functionally analogous to those produced by their host plants, particularly in their potential antiproliferative against *T. cruzi*. In this regard, this study aims to evaluate the trypanocidal activity of crude extracts obtained from these endophytic fungi, focusing on epimastigote and amastigote forms of *T. cruzi*, and cytotoxicity in J774A.1 macrophages. Among the characterized endophytes, *Montagnula* sp. exhibited the highest trypanocidal activity, comparable to benznidazole. To our knowledge, this is the first report of trypanocidal activity in *Montagnula* sp., expanding current understanding of endophytic fungi as sources of novel therapeutic agents against CD.

## 2. Materials and Methods

### 2.1. Cell Lines and Parasites

Murine macrophages J774A.1 (ATCC TIB-67), kindly provided by the Cell and Functional Biology and Biomolecule Engineering Group (Universidad Antonio Nariño), were cultured in Dulbecco’s Modified Eagle Medium (DMEM, high glucose; Gibco, Baltimore, MD, USA), supplemented with 10% heat-inactivated fetal bovine serum (FBS), 100 U/mL penicillin, and 100 μg/mL streptomycin. Cultures were maintained at 37 °C in a humidified atmosphere containing 5% CO_2_.

Epimastigote forms of *T. cruzi* (Sylvio X10 strain, TcI), provided by the Fundación Cardiovascular de Colombia, were cultured in liver infusion tryptose (LIT) medium supplemented with 10% heat-inactivated FBS and incubated at 28 °C.

### 2.2. Endophytic Fungi from L. alba

The endophytic fungi evaluated in this study were isolated from healthy *Lippia alba* plants provided by local farmers from two municipalities in Santander Department, Colombia: Betulia (Santa Bárbara village) and El Playón (La Naranjera village). The plant material was collected as part of the research project “Growth of *Lippia alba* (Mill.) N.E.Br. and its associated fungal endophytes. A total of seven morphologically distinct endophytic fungal isolates were obtained and are currently deposited in the Biological Collection of the Universidad de Santander (CBUDES), Bucaramanga, Colombia.

The fungal isolates used in this study are deposited in the Biological Collection of the Universidad de Santander (CBUDES), Bucaramanga, Colombia, from which the strains used in this study were obtained.

A total of seven morphologically distinct *L. alba* endophytic fungi were obtained from the CBUDES collection. The fungi were initially replicated on potato dextrose agar (PDA; Oxoid, Basingstoke, UK) supplemented with chloramphenicol. Plates were incubated at room temperature and monitored daily. Subsequently, macroscopic characterization was performed based on colony morphology on PDA medium. Preliminary identification of fungal was based on macroscopic (colony morphology, pigmentation, and growth rate) and microscopic characteristics (hyphal structure, spore morphology, and septation) of cultures grown on PDA. The fungal isolates were gently pressed onto QIAcard FTA sample cards (Qiagen, Germantown, MD, USA) to allow sufficient transfer of material, allowed to dry at room temperature and stored in individual protected sleeves until further processing.

### 2.3. Molecular Identification of Endophytic Fungi

Genomic DNA was extracted from fungal material applied to QIAcard FTA sample cards using the FTA Purification Reagent (Whatman, GE Healthcare, Piscataway, NJ, USA), following the manufacturer’s instructions. Internal Transcribed Spacer (ITS) PCR products were generated using the primer pair ITS4/ITS5 [19]. PCR reactions were performed in 20 μL volumes containing 10 μL of Platinum™II Hot-Start PCR Master Mix (2×) (Thermo Fisher Scientific, Waltham, MA, USA) 0.4 μL of each primer (10 μM), 5–10 ng of template DNA (1–2 μL), and 7–8 μL of PCR-grade water. Amplification was performed in a C1000 Touch PCR Thermal Cycler (Bio-Rad, Hercules, CA, USA) using PCR cycle conditions described elsewhere [20]. Amplicons were bi-directionally sequenced using BigDye™3.1 Terminator Cycle sequencing kit on an Applied Biosystems SeqStudio Genetic Analyzer (Thermo Fisher Scientific, Waltham, MA, USA). Sequences were quality trimmed and assembled using CLC Main Workbench v.24 (Qiagen, Germantown, MD, USA) using default settings. The sequences obtained were compared with reference sequences deposited in the NCBI database using the BLASTn algorithm.

### 2.4. Preparation of Crude Extracts

Secondary metabolites were obtained by submerged fermentation. Ten agar plugs (8 mm diameter) from actively growing fungal cultures were inoculated into 100 mL of potato dextrose broth (PDB) contained in 250 mL Erlenmeyer flasks and incubated at 120 rpm for 15 days. After incubation, ethyl acetate was added to the culture broth at a 3:1 (*v*/*v*) ratio and the mixture was sonicated for 20 min. The culture was subsequently filtered through 0.45 μm membranes to remove mycelium. The organic phase was recovered and concentrated under reduced pressure using a rotary evaporator at 40 °C. Crude ethyl acetate extracts obtained from submerged potato dextrose broth (PDB) cultures of the endophytic fungi were stored at −20 °C and resuspended in phosphate-buffered saline (PBS) prior to biological assays.

### 2.5. Trypanocidal Activity

For epimastigotes assays, parasites (5 × 10^5^ parasites/mL) were seeded in 96-well plates containing LIT medium (Liver Infusion Tryptose) (Becton-Dickinson, Miami, FL, USA) and exposed to crude extracts at concentrations ranging from 7.4 to 200 μg/mL. Plates were incubated at 28 °C for 72 h. Untreated parasites and BNZ were used as negative and positive controls, respectively. Parasite growth was assessed by direct counting using a Neubauer chamber. Trypanocidal activity was expressed as IC_50_ values.

For amastigote assay, J774A.1 macrophages were seeded on chamber slides at a density of 3 × 10^4^ cells/mL and infected with *T. cruzi* trypomastigotes at a 1:5 cell-to-parasite ratio. After 24 h of incubation at 37 °C and 5% CO_2_, infected cells were treated with crude extracts (7.4–200 μg/mL) for an additional 24 h. Cells were stained using Wright’s stain, and infection rates were determined by counting at least 300 cells per condition. IC_50_ values were calculated based on the percentage of infected cells relative to untreated controls.

### 2.6. Cytotoxicity Assay

J774A.1 macrophages (4 × 10^4^ cells/mL) were seeded in 96-well plates and incubated for 24 h. Cells were then exposed to crude extracts (22.2–600 μg/mL) for 24 h. Cell viability was evaluated using the MTT assay (3-(4,5-dimethylthiazol-2-yl)-2,5-diphenyltetrazolium bromide). Absorbance was measured at 570 nm using a Multiskan Sky microplate reader (Thermo Fisher Scientific, Waltham, MA, USA). Cytotoxicity was expressed as half-maximal cytotoxic concentration (CC_50_).

### 2.7. Morphological and Mitochondrial Analysis

Morphological alterations in treated and untreated macrophages were evaluated by light microscopy following Wright staining. Mitochondrial membrane potential (Δψm) was assessed using the MitoProbe™ JC-1 assay (Invitrogen, Carlsbad, CA, USA), while mitochondrial superoxide production was measured using MitoSOX™ Red (Invitrogen), according to the manufacturer’s instructions. Fluorescence signals were analyzed using a Nikon Eclipse Ni-U fluorescence microscope (Nikon Eclipse Ni-U, Melville, NY, USA). Approximately 200 cells per condition were evaluated. Untreated and CCCP-treated cells were used as negative and positive controls, respectively.

### 2.8. Statistical Analysis

All experiments were performed in triplicate and independently repeated three times. IC_50_ and CC_50_ values were calculated using nonlinear regression with XLfit5™ software 5.5.0.5. (IDBS, Boston, MA, USA). Normality and homoscedasticity were assessed using the Shapiro–Wilk and Levene tests, respectively. Statistical significance was determined by one-way ANOVA followed by Dunnett’s post hoc Statistical significance was determined by one-way ANOVA followed by Dunnett’s post hoc test using GraphPad Prism 9.5.1 (Dotmatics, Boston, MA, USA). The *p*-value < 0.05 was considered statistically significant.

### 2.9. UHPLC-ESI-HRMS Analysis

The profiling and quantification of phenolic compounds in the ethyl acetate extract of *Montagnula* sp. exhibiting the highest trypanocidal activity were conducted using ultra high-performance liquid chromatography coupled to electrospray ionization high resolution mass spectrometry (UHPLC-ESI-HRMS). The solid extract was dissolved in a mixture of methanol and water (1:1, *v*/*v*) containing 0.2% formic acid to yield a final concentration of 1 mg/mL. The mixture was vortexed for 5 min, sonicated for 20 min, and an aliquot of 2 μL was directly injected into the UHPLC system. Chromatographic separation was performed on a Dionex Ultimate 3000 UHPLC system (Thermo Scientific, Sunnyvale, CA, USA) equipped with a Hypersil GOLD Aq column (100 × 2.1 mm, 1.9 μm). The mobile phase consisted of water (A) and methanol (B), both containing 0.1% formic acid and 5 mM ammonium formate. The gradient elution was programmed as follows: 0–8 min, linear transition from 0% to 100% B; 8–12 min, isocratic hold at 100% B; 12–13 min, return to initial conditions (0% B). The flow rate was maintained at 0.3 mL/min. The UHPLC system was coupled to a high resolution Orbitrap mass spectrometer equipped with an electrospray ionization (ESI) source, operating in positive electrospray ionization mode. The capillary voltage was set to 3.5 kV. Data acquisition was performed in full scan mode over a mass range of *m*/*z* 80–1000, targeting protonated molecules [M + H] ^+^. Compound identification was confirmed by evaluating isotopic patterns and fragmentation profiles, achieving a mass accuracy of <1 ppm. The quantification of the target analytes was performed using external calibration curves generated with certified reference standards: caffeic acid, ferulic acid, naringenin, apigenin, and ursolic acid all purchased from Sigma-Aldrich (Sigma-Aldrich, 200 St. Louis, MO, USA), with purities ≥ 99%.

## 3. Results

### 3.1. Molecular Identification of the Endophytic Fungal Isolates and Phylogenetic Analysis of Montagnula sp. Strain HELA-A

**Figure 1:**
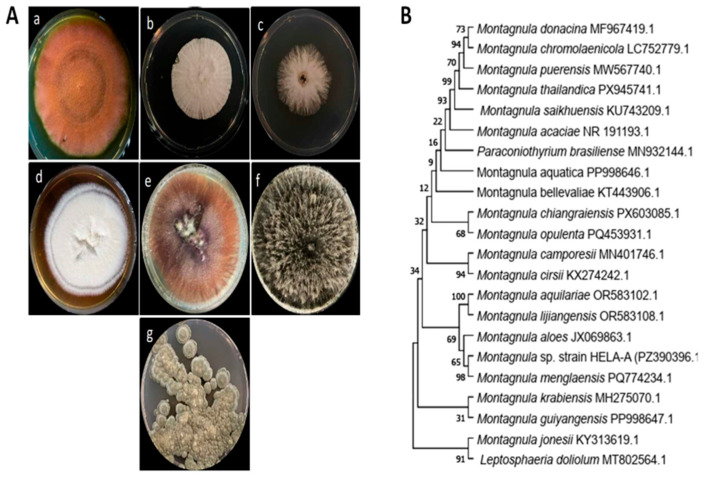
Morphological and molecular characterization of the endophytic fungal isolates recovered from *Lippia alba*. (**A**) Colony morphology of the seven endophytic fungal isolates grown on PDA medium: (**a**) *Montagnula* sp. isolate HELA-A; (**b**) *Xylaria* sp. isolate HELA-B; (**c**) *Xylaria* sp. isolate HELA-C; (**d**) *Periconia* sp. isolate HELA-D; (**e**) *Fusarium* sp. isolate HELA-E; (**f**) *Lasiodiplodia* sp. isolate HELA-F; and (**g**) *Penicillium* sp. isolate HELA-G. (**B**) Maximum-likelihood phylogenetic tree based on ITS sequences showing the phylogenetic placement of *Montagnula* sp. strain HELA-A among representative *Montagnula* species. Bootstrap values greater than 50% are indicated at the nodes. *Leptosphaeria doliolum* and *Paraconiothyrium brasiliense* were used as outgroup taxa.Morphological and molecular characterization of the endophytic fungal isolates recovered from *Lippia alba*.

A total of seven morphologically distinct *L. alba* endophytic fungi were obtained from the Biological Collection of the Universidad de Santander (CBUDES), Colombia. Four strains originated from leaves *Montagnula* sp. isolate HELA-A, *Xylaria* sp. isolate HELA-B, *Periconia* sp. isolate HELA-D, and *Penicillium* sp. isolate HELA-G, two from stems *Xylaria* sp. isolate HELA-C and *Lasiodiplodia* sp. isolate HELA-F, and one from roots *Fusarium* sp. isolate HELA-E. Preliminary identification based on colony morphology suggested affiliation with the phylum Ascomycota (Figure 1A). Molecular identification using ITS sequence analysis confirmed the taxonomic assignment at the genus level, including *Montagnula* sp. isolate HELA-A, *Xylaria* sp. isolate HELA-B, *Periconia* sp. isolate HELA-D, *Penicillium* sp. isolate HELA-G, *Xylaria* sp. isolate HELA-C, *Lasiodiplodia* sp. isolate HELA-F, and *Fusarium* sp. isolate HELA-E (Table 1). The fungal strains were subsequently subjected to submerged fer-mentation for crude extract production. To further support the taxonomic placement of the isolate exhibiting the highest trypanocidal activity (HELA-A), a maximum-likelihood phylogenetic analysis based on ITS sequences was performed using representative Montagnula species retrieved from GenBank, together with appropriate outgroup taxa. The analysis placed strain HELA-A within the Montagnula clade, clustering most closely with *M. menglaensis* with strong bootstrap support (98%), confirming its affiliation with the genus. However, considering the limited species-level resolution of the ITS marker within Montagnula, the isolate was conservatively designated as *Montagnula* sp. strain HELA-A (Figure 1B).

### 3.2. Trypanocidal Activity and Cytotoxicity of Endophytic Fungal Extracts

**Figure 2:**
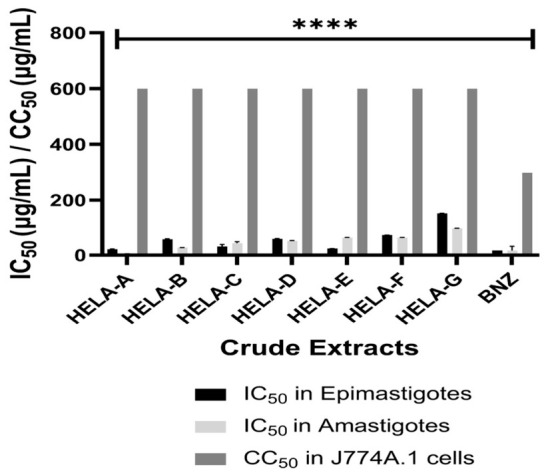
Trypanocidal activity against *T. cruzi* and cytotoxicity on murine macrophages cells (J774A.1) of *Lippia alba* endophytic fungi crude extracts. IC_50_: Inhibitory Concentration 50; CC_50_: Cytotoxic Concentration 50; SI, selectivity index (CC_50_/IC_50_). Values are expressed as mean ± standard deviation (SD) of three independent experiments; HELA: *Lippia alba* endophytic fungus; *Montagnula* sp. isolate HELA-A; *Xylaria* sp. isolate HELA-B; *Xylaria* sp. isolate HELA-C; *Periconia* sp. isolate HELA-D; *Fusarium* sp. isolate HELA-E; *Lasiodiplodia* sp. isolate HELA-F and (g) *Penicillium* sp. isolate HELA-G; BNZ: Benznidazole; **** *p* <0.0001.

The trypanocidal activity of crude ethyl acetate extracts from seven *L. alba* endophytic fungi obtained from the CBUDES collection was evaluated against epimastigote and amastigote forms *T. cruzi*. Cytotoxicity was assessed in uninfected J774A.1 murine macrophages to calculate selectivity indices. All extracts, except that obtained from *Penicillium* sp. (HELA-G), inhibited epimastigote growth at concentrations below 200 μg/mL (Table 2). The extract derived from *Montagnula* sp. (HELA-A) exhibited the highest activity against epimastigotes, with an IC_50_ value of 22.1 ± 0.5 μg/mL, followed by *Xylaria* sp. (HELA-B; IC_50_ = 23 ± 0.7 μg/mL), and *Fusarium* sp. (HELA-E; IC_50_ = 23 ± 0.7 μg/mL). In contrast, the *Penicillium* sp. extract showed the lowest activity (IC_50_ = 152 ± 23 μg/mL). The most active extracts against epimastigote forms were further evaluated against intracellular amastigotes. HELA-A and HELA-E inhibited amastigote proliferation with IC_50_ values of 29.2 μg/mL and 43.2 μg/mL, respectively, whereas BNZ exhibited an IC_50_ value of 34.2 μg/mL under the same experimental conditions (Table 2 and Figure 2).

Cytotoxicity assays indicated that all crude extracts displayed low toxicity toward J774A.1 macrophages, with CC_50_ values higher than 600 μg/mL. In contrast, BNZ showed a CC_50_ value of 298 ± 1.9 μg/mL. Selectivity indices calculated for the most active extracts ranged from 4 to 26, with HELA-A exhibiting the highest selectivity index (Table 2).

### 3.3. Effects of Selected Extracts on Macrophage Morphology and Mitochondrial Function

**Figure 3:**
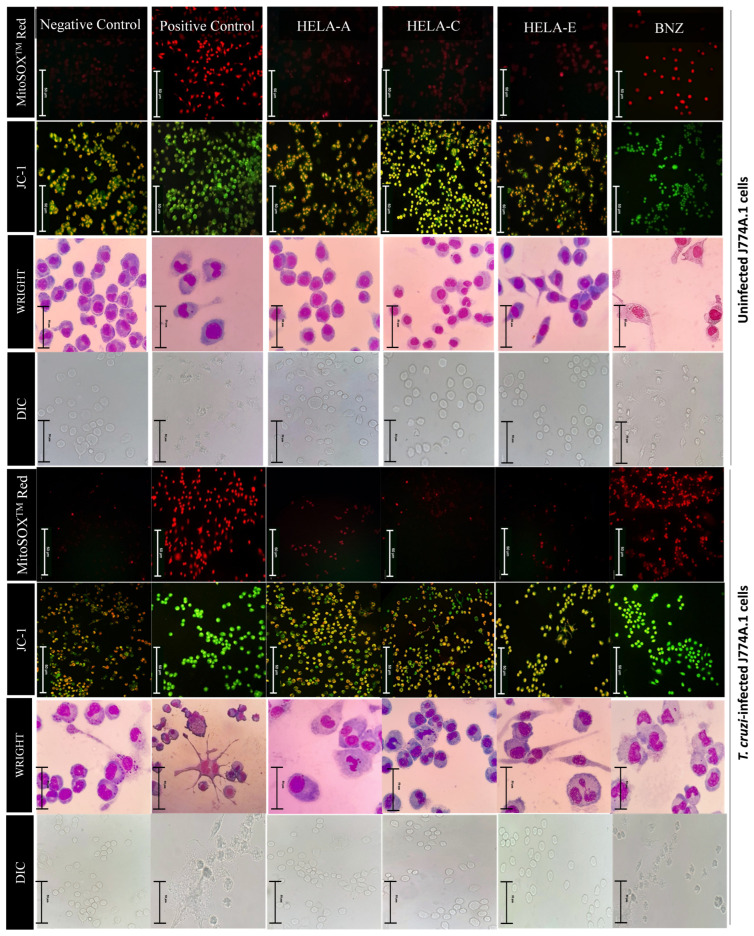
Fluorescence analysis of mitochondrial superoxide production and membrane potential in *T. cruzi*-infected J774A.1 macrophages following treatment with selected crude extracts. Negative control: untreated cells; Positive control: CCCP-treated cells; HELA: *L. alba* endophytic fungi extracts; BNZ: Benznidazole. MitoSOX™ Red (40×), JC-1 (40×), Wright (100×), and DIC (differential interference contrast) images as representative from ten consecutive microscopic fields.

The effects of selected crude extracts on uninfected and *T. cruzi*-infected J774A.1 macrophages were evaluated using optical and fluorescence microscopy. Analyses were performed using the three extracts that exhibited the highest trypanocidal activity: HELA-A, HELA-C, and HELA-E, tested at their respective IC_50_ and CC_50_ concentrations. Untreated, uninfected macrophages exhibited normal morphophysiological characteristics, including intact cell morphology, preserved plasma membrane integrity, and strong adherence, as observed by Wright staining and differential interference contrast (DIC) microscopy (Figure 3). These cells showed low mitochondrial superoxide production, indicated by weak MitoSOX™ Red fluorescence (Invitrogen, Thermo Fisher Scientific, Eugene, OR, USA), and preserved mitochondrial membrane potential, evidenced by predominant yellow JC-1 fluorescence. Similarly, macrophages treated with HELA-A and HELA-C extracts displayed morphological features comparable to those of untreated control cells, without evident loss of membrane integrity or cell detachment (Figure 3). MitoSOX™ Red fluorescence intensity remained low, and JC-1 staining indicated preserved mitochondrial membrane potential. In contrast, HELA-E-treated cells exhibited mild cellular aggregation and discrete alterations in cell morphology, accompanied by a partial decrease in JC-1 fluorescence.

On the other hand, cells exposed to positive control treatments (CCCP or DMSO) showed marked morphological alterations, including cell swelling, loss of adhesion, and membrane disruption (Figure 3). These alterations were accompanied by intense MitoSOX™ Red fluorescence and a predominance of green JC-1 fluorescence, indicating increased mitochondrial superoxide production and loss of mitochondrial membrane potential. Untreated *T. cruzi*-infected macrophages exhibited increased MitoSOX™ Red fluorescence compared with uninfected controls, together with a moderate reduction in JC-1 fluorescence (Figure 3). Treatment with HELA-A produced fluorescence patterns similar to those observed in untreated infected macrophages, displayed reduced JC-1 fluorescence and mild morphological alterations. HELA-C-treated infected cells showed cellular aggregation while maintaining JC-1 fluorescence.

BNZ-treated infected macrophages exhibited pronounced morphological damage, including loss of membrane integrity and nuclear alterations, accompanied by increased MitoSOX™ Red fluorescence and near-complete loss of JC-1 signal (Figure 3).

### 3.4. Chemical Characterization of the Montagnula sp. Extract by UHPLC-ESI-HRMS

The ethyl acetate extract of *Montagnula* sp., which exhibited the highest trypanocidal activity, was chemically characterized by UHPLC-ESI-HRMS. The chromatographic analysis revealed well-resolved peaks within a total run time of 13 min, allowing the detection and quantification of several phenolic and terpenoid compounds. Five major compounds were identified based on accurate mass measurements (Δppm < 1), retention times (RT), isotopic patterns, and comparison with authentic standards when available. These included caffeic acid (RT 3.9 min, *m*/*z* 179.03), ferulic acid (RT 5.2 min, *m*/*z* 195.06), naringenin (RT 6.0 min, *m*/*z* 273.07), apigenin (RT 6.5 min, *m*/*z* 271.06), and ursolic acid (RT 9.4 min, *m*/*z* 457.37). Ferulic acid was the most abundant compound detected, with a concentration of 14.9 mg/kg, followed by caffeic acid (4.2 mg/kg). Naringenin, apigenin, and ursolic acid were present at concentrations of 1.3 mg/kg, 1.2 mg/kg, and 0.9 mg/kg, respectively (Table 3). Other compounds, including xanthines, catechins, anthocyanins, and various phenolic acids and flavonoids, were detected below the limit of quantification (LOQ), indicating they were present only in trace amounts.

## 4. Discussion

In this study, crude extracts from endophytic fungi from different tissues of *L. alba* exhibited variable trypanocidal activity against *T. cruzi*. Most extracts inhibited epimastigote growth, while a subset also showed activity against intracellular amastigotes, underscoring the potential of endophytic fungi as sources of antiparasitic agents. Among the evaluated extracts, the crude extract of *Montagnula* sp. isolate HELA-A consistently exhibited the highest activity against both parasite forms, with IC_50_ values comparable to those of benznidazole (BNZ). The phylogenetic analysis further supported the taxonomic placement of HELA-A within the genus Montagnula, providing additional confidence in the taxonomic identity of the bioactive isolate despite the limited species-level resolution of the ITS marker. This extract combined potent antitrypanosomal activity with low cytotoxicity toward host cells, suggesting that *Montagnula* sp. represents a promising and previously unexplored source of bioactive metabolites against *T. cruzi*. To our knowledge, this is the first report describing the antitrypanosomal activity of an endophytic Montagnula species.

A key finding was the high selectivity of the most active endophytic extracts. While BNZ induced considerable cytotoxicity in J774A.1 macrophages, all fungal extracts exhibited CC_50_ values above 600 μg/mL, yielding favorable selectivity indices, particularly for HELA-A. The high selectivity indicated by the selectivity index was further supported by morphophysiological and mitochondrial analyses of macrophages treated with the *Montagnula* sp. extract. Unlike BNZ, which was associated with mitochondrial dysfunction, oxidative stress, and necrotic changes in infected macrophages, HELA-A preserved cellular morphology, maintained mitochondrial membrane potential, and did not markedly increase superoxide production. These observations suggest that the trypanocidal activity is achieved without overt host-cell damage, a highly desirable feature for antichagasic candidates. Recent evidence indicates that infection-induced mitochondrial ROS can facilitate intracellular *T. cruzi* proliferation, whereas antioxidant or mitochondria-targeted ROS inhibition reduces parasite burden, supporting the idea that limiting oxidative stress while preserving host-cell integrity may contribute to the selective antiparasitic activity observed for the HELA-A extract [21].

UHPLC–ESI–HRMS analysis of the *Montagnula* sp. extract tentatively identified phenolic compounds (caffeic acid, ferulic acid, naringenin, apigenin) together with the terpenoid ursolic acid. These findings are consistent with previous reports describing phenolic and terpenoid metabolites produced by endophytic fungi as compounds with potential antiparasitic and cytoprotective properties. Likewise, terpenes such as limonene, citral, and caryophyllene oxide have been widely recognized for selective trypanocidal and cardioprotective activity in murine models of chagasic cardiomyopathy [8,22], effects attributed to their immunomodulatory properties and dual pro-oxidant/antioxidant behavior. This dual redox activity has also been described for plant-derived polyphenols, which can shift between antioxidant and pro-oxidant states depending on concentration, metal-reducing potential, chelating capacity, pH, and solubility [22]. Recent studies further demonstrate that phenolic acids modulate macrophage inflammatory responses and redox pathways while exerting significant trypanocidal effects [23]. Notably, Vargas-Munévar et al. [23] reported that microencapsulated extracts of *Theobroma cacao*, enriched in catechins and epicatechins, displayed superior anti-*T. cruzi* activity compared to BNZ, alongside cardioprotective effects against oxidative stress, inflammation, and cell death. The selective antiproliferative action of these polyphenolic extracts was attributed to their pronounced dual pro-oxidant/antioxidant properties. Although the identified classes of metabolites have been associated with antiparasitic activity in previous studies, the present work evaluated crude extracts; therefore, the individual contribution of each compound or potential synergistic interactions cannot be established. Future bioactivity-guided fractionation coupled with the evaluation of purified compounds will be necessary to identify the metabolites responsible for the observed antitrypanosomal activity.

Phenolic compounds such as caffeic acid and ferulic acid are well recognized as dual agents, acting as pro-oxidants in highly metabolically active cells, such as parasites while exerting antioxidant effects in host tissues [23,24,25,26]. Interestingly, both were identified herein in the *Montagnula* sp. extract. Caffeic acid was identified at RT 3.9 min with *m*/*z* of 179.03. This polar hydroxycinnamic acid, composed of a benzene ring with two *ortho*-hydroxyl groups and a propanoic acid chain, exhibits strong antioxidant properties. Its conjugated double bond enhances stability and reactivity in redox processes [24]. Pharmacologically, caffeic acid has been reported as a potent inhibitor of *T. cruzi* α-carbonic anhydrase (TcCA) with an inhibitory constant (KI) of 1.8 μM, positioning it as a promising anti-Chagas candidate [25]. Ferulic acid, identified at RT 5.2 min (*m*/*z* 195.06), is a methoxylated hydroxycinnamic acid, differing from caffeic acid by the presence of a methoxy (-OCH_3_) group on the aromatic ring. This substitution increases lipophilicity and enhances radical scavenging activity [27]. Biologically, ferulic acid regulates macrophage polarization via ROS/NF-κB signaling [26]. Naringenin, detected at RT 6.0 min (*m*/*z* 273.07), is a flavanone-type flavonoid, characterized by a chromanone ring with two phenolic rings and three hydroxyl groups that confer antioxidant capacity. The absence of a C2-C3-double bond distinguishes it as a flavanone, contributing to its notable antioxidant and anti-inflammatory properties [28]. Recent studies highlight its substantial trypanocidal activity against *T. cruzi* [29]. Apigenin, another flavonoid identified at RT 6.5 min (*m*/*z* 271.06), belongs to the flavone-type flavonoid. Structurally similar to naringenin, it possesses a C2-C3 double bond in the C-ring, two phenolic rings, and three hydroxyl groups, conferring strong antioxidant activity [28]. Ursolic acid, the sole terpene detected in the organic extract of *Montagnula* sp., appeared as a prominent peak at RT 9.4 min (*m*/*z* 457.37). This pentacyclic triterpenoid, composed of five fused rings and a carboxylic acid group has been reported to induce autophagic and xenophagic responses in *T. cruzi*-infected cells [23], facilitating parasite clearance while preserving host-cell viability. Together, the phenolic and terpenoid metabolites identified may underlie the balanced phenotypic response observed in macrophages treated with the *Montagnula* extract, and their coexistence may contribute to the observed trypanocidal activity [29]. Overall, extracts from *Montagnula* sp. and *Fusarium* sp. displayed the strongest inhibitory effects against both epimastigote and amastigote forms of *T. cruzi*, with activity comparable to BNZ. The *Montagnula* sp. extract combined potent antiparasitic activity with low cytotoxicity, high selectivity, and preservation of host-cell integrity. While these findings are promising, further studies involving bioassay-guided fractionation, purification of active metabolites, and in vivo evaluation are required to fully establish the therapeutic potential of *Montagnula* sp. and other endophytic fungi identified herein. Beyond pharmacological relevance, this study underscores the biotechnological potential of endophytic fungi as sustainable platforms for metabolite production. Endophytes can synthesize bioactive compounds identical or analogous to those of their host plants, offering advantages in sustainability, scalability, and compositional consistency compared to direct plant extraction. In the case of *L. alba*, whose essential oils exhibit strong trypanocidal and cardioprotective effects [8,22] but suffer from compositional variability [30], endophytic fungi may represent a more controllable and reliable alternative for metabolite production.

## 5. Conclusions

This study provides the first evidence of trypanocidal activity associated with endophytic fungi from *Lippia alba* and identifies *Montagnula* sp. as a promising and previously unexplored fungal source for anti-*Trypanosoma cruzi* drug discovery. These findings highlight the potential of medicinal plant-associated endophytic fungi as sustainable and scalable platforms for the development of new therapeutic agents against Chagas disease. Future studies involving bioassay-guided fractionation, the isolation of active metabolites, and in vivo validation will be essential to further define their pharmacological potential.

## Figures and Tables

**Table 1:** Molecular identification of endophytic fungal isolates from *Lippia alba* based on ITS sequence analysis.

Code	Host Plant Tissue	Closest Blast Match (NCBI)	Identity (%)	Accession Number
HELA-A	Leaf	OR583111	98.7	PZ390396
HELA-B	Leaf	HQ022442	99.2	PZ390399
HELA-C	Stalk	JQ760826	99.7	PZ390397
HELA-D	Leaf	MW063162	100	PZ390401
HELA-E	Root	GQ505755	100	PZ390400
HELA-F	Stalk	NR_111264	100	PZ390398
HELA-G	Leaf	ON426869	100	PZ390395

HELA: *Lippia alba* endophytic fungus.

**Table 2:** Trypanocidal activity, cytotoxicity, and selectivity indices of crude extracts from *Lippia alba* endophytic fungi.

Extract Code	Fungal Species	IC_50_ Epimastigotes ± SD (μg/mL)	Amastigotes IC_50_ ± SD (μg/mL)	SI	J774A.1 CC_50_ ± SD (μg/mL)
HELA-A	*Montagnula* sp.	22.1 ± 0.5	29.2 ± 3.0	21	>600
HELA-B	*Xylaria* sp.	60.0 ± 2.0	68.3 ± 6.2	9	>600
HELA-C	*Xylaria* sp.	36.0 ± 1.4	46.3 ± 3.0	13	>600
HELA-D	*Periconia* sp.	62.2 ± 0.5	66.2 ± 3.9	9	>600
HELA-E	*Fusarium* sp.	23.0 ± 0.7	43.2 ± 1.7	14	>600
HELA-F	*Lasiodiplodia* sp.	75.0 ± 3	74.0 ± 6.0	8	>600
HELA-G	*Penicillium* sp.	152.0 ± 23	134.3 ± 1.2	4	>600
BNZ	*-*	17.0 ± 0.9	34.2 ± 5.1	18	298.0 ± 1.9
Ethyl acetate	*-*	>200	>200	3	>600 ± 0

IC_50_: 50% inhibitory concentration; CC_50_: 50% cytotoxic concentration; SI: selectivity index (CC_50_/IC_50_). Values are expressed as the mean ± standard deviation (SD) of three independent experiments. HELA: *Lippia alba* endophytic fungus; BNZ: benznidazole. *Montagnula* sp. isolate HELA-A; *Xylaria* sp. isolate HELA-B; *Xylaria* sp. isolate HELA-C; *Periconia* sp. isolate HELA-D; *Fusarium* sp. isolate HELA-E; *Lasiodiplodia* sp. isolate HELA-F; and *Penicillium* sp. isolate HELA-G.

**Table 3:** UHPLC-ESI-HRMS chromatogram of the ethyl acetate extract of *Montagnula* sp.

RT (min)	Abund. Area	Fragm. (*m*/*z*)	Compound * Formula ** MW	Concentration (mg/kg)	Chemical Structure
3.9	7.0 × 10^7^	179.03	Caffeic acid (HO)_2_C_6_H_3_CH=CHCO_2_H 180.16 g/mol	4.2	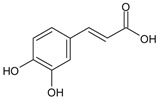
5.2	1.6 × 10^8^	195.06	Ferulic acid HOC_6_H_3_(OCH_3_) CH=CHCO_2_H 194.18 g/mol	14.9	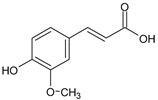
6.0	1.2 × 10^6^	273.07	Naringenin C_15_H_12_O_5_ 272.25 g/mol	1.3	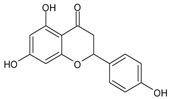
6.5	2.3 × 10^7^	271.06	Apigenin C_15_H_10_O_5_ 270.24 g/mol	1.2	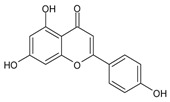
9.4	1.4 × 10^6^	457.37	Ursolic acid C_30_H_48_O_3_ 456.70 g/mol	0.9	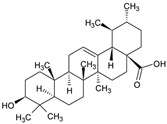

RT: Retention time; Abund. area: Abundance area in aleatory units; Fragm.: Fragmentation; * Identified compound; ** Empirical formula; MW: Molecular weight.

## Data Availability

The data supporting the findings of this study are available within the article. The ITS sequence of *Montagnula* sp. strain HELA-A has been deposited in GenBank under accession number PZ390396. Additional data are available from the corresponding author upon reasonable request.
